# Early Auditory Temporal Processing Deficit in Children with Autism Spectrum Disorder: The Research Domain Criteria Framework

**DOI:** 10.3390/brainsci14090896

**Published:** 2024-09-03

**Authors:** Atoosa Sanglakh Ghoochan Atigh, Mohammad Taghi Joghataei, Shadi Moradkhani, Mehdi Alizadeh Zarei, Mohammad Ali Nazari

**Affiliations:** 1 Department of Neuroscience, Faculty of Advanced Technologies in Medicine, Iran University of Medical Sciences, Tehran 1449614535, Iran; atoosa.atigh@gmail.com (A.S.G.A.); mt.joghataei@yahoo.com (M.T.J.); 2 Department of Neuroscience, Faculty of Psychology and Educational Science, University of Tabriz, Tabriz 5166616471, Iran; shadi.moradkhani@gmail.com; 3 Occupational Therapy Department, School of Rehabilitation Sciences, Iran University of Medical Sciences, Tehran 1545913487, Iran; mehdii.alizadeh@yahoo.com

**Keywords:** autism spectrum disorder (ASD), biomarker, electrophysiology, time processing, ISI deviant, mismatch negativity (MMN)

## Abstract

Altered sensory processing especially in the auditory system is considered a typical observation in children with autism spectrum disorder (ASD). Auditory temporal processing is known to be impaired in ASD children. Although research suggests that auditory temporal processing abnormalities could be responsible for the core aspects of ASD, few studies have examined early time processing and their results have been conflicting. The present event-related potential (ERP) study investigated the early neural responses to duration and inter-stimulus interval (ISI) deviants in nonspeech contexts in children with ASD and a control group of typically developing (TD) children matched in terms of age and IQ. A passive auditory oddball paradigm was employed to elicit the mismatch negativity (MMN) for change detection considering both the duration and ISI-based stimulus. The MMN results showed that the ASD group had a relatively diminished amplitude and significant delayed latency in response to duration deviants. The findings are finally discussed in terms of hyper-hyposensitivity of auditory processing and the fact that the observed patterns may potentially act as risk factors for ASD development within the research domain criteria (RDoC) framework.

## 1. Introduction

Autism spectrum disorder (ASD) is an early-onset neurodevelopmental disorder that is characterized by diminished social communication, repetitive patterns of restricted behaviors and interests [1,2,3], and extreme resistance to change [4,5]. Despite impairments in social communication, some reports point to enhanced auditory and visual perceptual processing in individuals with autism [6]. For example, behavioral and neurophysiological investigations have shown that individuals with autism might display shortcomings in perceiving sound durations [7,8]. Aside from duration effects, another critical variable that varies between studies is the inter-stimulus interval (ISI), which refers to the period between stimulus offset and the onset of the following stimulus [9,10]. The ISI has been shown to be crucial in optimizing auditory responses [11]. The two characteristics of stimuli, specifically their duration and the interstimulus interval (ISI), have been reported to contribute to the representations used for higher-order cognition, such as speech perception [12,13]. Thus, considering these various aspects of temporal processing is essential for effectively investigating their neural mechanisms. In ASD, impaired temporal processing affects speech perception and audiovisual binding [14]. Additionally, impaired temporal processing may underlie core features of the disorder, including deficits in social cognition [15]. The atypical duration perception findings also indicate that children with autism employ distinct perceptual strategies compared to TD children for essential temporal cues crucial in speech recognition [16,17]. Understanding the reasons behind atypical auditory processing and its effects on speech perception and communication in autism is theoretically and practically significant due to the fundamental role of sensory perception in early development [18,19,20].

Moreover, the application of electrophysiological techniques such as event-related potentials (ERPs) has been proposed as a valuable avenue for investigating both the overt and covert processes underlying observed behavior, including their potential utility in diagnostic purposes [21,22]. Demonstrating ERP differences in auditory timing in children with autism compared to TD children contributes to our understanding of the relevant neural mechanisms [23,24]. The Diagnostic and Statistical Manual of Mental Diseases (DSM) classification system’s limitations in integrating recent progress in genetics, behavioral science, and neuroscience have impeded a comprehensive understanding of pathology [25]. Contrary to classical methods of classifying psychiatric disorders, diagnostic classification frameworks for mental disorders such as the Research Domain Criteria (RDoC) aim to incorporate neuroscience-based psychiatric classification by including neurobiological factors alongside clinical symptoms, especially in conditions like ASD [26,27]. Adopting this approach could open the door to developing more personalized treatment plans that address specific areas of vulnerability [27]. Any knowledge of auditory time processing is valuable for two primary reasons. Firstly, it provides insights into the workings of the typical auditory system when we examine how specific deficits such as ASD impact auditory time processing. Secondly, this knowledge about the auditory system is crucial for diagnosing and developing effective interventions. An important issue in this research line is determining whether time processing in autism is impaired at the early stages of auditory perception.

Mismatch negativity (MMN) is an ERP component that reflects early-stage processing of auditory timing without attention or behavioral tasks [28]. MMN represents the automatic detection of auditory changes in the human auditory and frontal cortices, triggering involuntary attention redirection toward novel or deviant stimuli [29]. Introducing deviant stimuli among a sequence of repetitive standard auditory stimuli results in the MMN component occurring between 150 and 250 milliseconds following the onset of the deviant stimulus [19]. Additionally, MMN amplitude and latency closely reflect an individual’s active discrimination performance, even when they are not paying attention [30]. Therefore, MMN serves as a valuable tool for investigating cortical auditory discrimination in clinical populations, including those with autism. The MMN component is computed by considering the difference between the amplitude of the negative peak elicited by a standard stimulus and the corresponding peak triggered by a deviant stimulus [31]. The amplitude of the MMN is associated with the extent of stimulus alteration [32,33]. This early ERP component has enabled the objective evaluation of the impact of different neuropsychiatric pathologies on involuntary attention. Furthermore, the MMN has been proposed as an alternative measure for the early detection of several diseases and the tracking of disorders’ progression [34,35,36,37].

MMN has recently been intensively used for investigating auditory perception in ASD [38,39]. Individuals with this developmental disorder have been described as lacking variation in tone, resembling a mechanical or monotonous intonation [40,41]. Various abnormalities in these responses have been observed in both children and adults with autism, indicating impaired sound-feature encoding [42]. Furthermore, research that employed pure tone stimuli in ASD reported divergent findings [43,44,45]. For example, Gomot et al. (2011) report that children with ASD had larger MMN responses to deviant stimuli. However, another study on low-functioning individuals with ASD reported reduced MMN amplitude [45]. It has been reported that children with autism exhibit diminished MMN for duration changes, which is significant only for non-speech changes [30]. Results of research investigating auditory processes in ASD individuals provide evidence that there is also an abnormality in temporal processing [46,47]. Vlaskamp et al. aimed to explain some of the variations in MMN findings in ASD by examining how individuals with ASD respond to duration deviants in an oddball task [43]. The authors found that individuals with ASD exhibited decreased MMN amplitude (at frontocentral electrodes: Cz and Fz) when exposed to duration deviants. These findings could indicate a lessened ability to detect duration alterations in auditory stimuli. The diminished MMN amplitudes observed in individuals with ASD may indicate a relative insensitivity to auditory stimuli, aligning with the hypothesis that hypo-responsiveness to sensory input is more prevalent in ASD than hyper-responsiveness [48,49]. Neurophysiologically, hyper-responsiveness and hypo-responsiveness correspond to increased or decreased neural firing, respectively [4]. To a certain extent, the discrepancies in ASD research can be attributed to variations in research methods. Some studies employed visual MMN paradigms, while others utilized auditory verbal stimuli as both standard and deviant, and still, others focused on deviants related to pitch, frequency, or duration [50,51]. Thus, MMN amplitudes and latencies might highly depend on the nature of the deviance (e.g., variations in frequency, duration, or pitch) between the deviant and standard stimuli, as well as whether the stimuli are pure tones or speech sounds. Additionally, these factors are influenced by the process of development. Another key question is whether atypical auditory processing in autism represents a domain-general perceptual deviation or a domain-specific change in sensitivity shaped by language experience. In a recent work, a pure tone condition and a vowel condition were used in an oddball paradigm. The MMN results showed that the autism group had diminished response amplitudes and delayed latency in the pure tone condition compared to the TD group, whereas no group difference was found in the vowel condition [52]. These findings suggest that atypical auditory processing in autism may reflect a domain-general perceptual deviation, as the deficits were apparent in pure sounds but not in language-specific stimuli. This supports the idea that the perceptual challenges in autism extend beyond language and are indicative of a broader auditory processing impairment.

Besides the methodological differences in previous works, the heterogeneity of ASD has also contributed to these inconsistencies [53,54]. The present research aimed to address these inconsistencies and obtain a more precise biomarker for children with ASD. We controlled several crucial factors in our study, including medication status and comorbid psychiatric conditions such as ADHD comorbidity, and ensured a matched group based on IQ, age, gender, and sensory profile among our participants. In this regard, we used an oddball paradigm to assess the automatic discrimination of sound duration and inter-stimulus interval (ISI) in two blocks as reflected by the MMN component amplitude and latency. Paradigms using such a combination of temporal deviants might enhance our understanding of sensory and timing processes in children with ASD. Our main question of this study was finding the different durations and ISIs of auditory beeps compared with TD control children. Building on the suggestion that individuals with ASD exhibit a relative insensitivity to auditory stimuli, which aligns with the prevalence of hypo-responsiveness to sensory input in this population, we anticipated observing reduced MMN amplitude and latency for duration changes. Additionally, given the critical role of ISI in optimizing auditory responses and enhancing speech recognition, we hypothesized that individuals with ASD would demonstrate a relative hypo-sensitivity to ISI deviants in MMN characteristics. This dual-focus approach aimed to shed additional light on temporal processing mechanisms in ASD, providing an understanding of auditory perception in this population.

## 2. Materials and Methods

### 2.1. Participants

The participants were selected from children aged 4.5–11 years old. The TD controls were recruited through social media and online forms. Participants with ASD were introduced from the Arman-Shayan clinic in Tehran, Iran. A proficient clinician who is a specialist in ASD performed all psychiatric examinations based on the DSM-5′s criteria for autism [3]. The first sample consisted of 35 ASD and 35 TD control children.

The Gilliam Autism Rating Scale (GARS-3) questionnaire was used to identify children with ASD [55]. The GARS-3, translated and adapted for Iranian children, was previously validated with high sensitivity (99%) and specificity (100%) [56]. Children with mental impairment or head injuries were excluded from the study. All recruited ASD children were drug-naïve. In the first screening interviews, the individuals with comorbid psychotic diagnoses or a history of neurological pathology were enlisted. The Conners Comprehensive Behavior Rating Scales (CBRS), parent form, was utilized to assess all participants for ADHD symptoms to exclude them from the study [57]. The Persian version of the CBRS–Parents’ Form exhibited proper reliability and validity for screening the Iranian pediatric population [58]. Three ASD children with a comorbidity of ADHD were excluded. Participants with excessive movements or tics (motor, vocal, etc.) were also excluded. We used the tactile section from the Sensory Profile to evaluate tactile sensitivity and selected those participants who did not exhibit hypersensitivity to tactile stimulation [59]. In addition, children with ASD who exhibited a heightened level of tactile sensitivity, which prevented the recording of EEG, were not included in the study. The tactile sensitivity of participants was evaluated based on their ability to tolerate the EEG recording process.

Thus, eleven TD children and fifteen others with ASD showed limited cooperation during the data recording process and displayed a high level of movement, leading to their exclusion from the study. Finally, 44 participants aged 4.5–11 years were included in this study. Participants included children with ASD (*n* = 20, male: 80%, mean age: 6.6 years, SE = 0.526) and TD controls (*n* = 24, male: 71%, mean age: 7.12 years, SE = 0.393).

All participants were matched based on IQ, age, and handedness. The IQ of the children was assessed by Raven, and we recruited children in the Normal IQ range (90–110) (ASD: mean = 102.10, SE = 1.488; TD controls: mean = 103.96, SE = 1.214) [60]. Using the Sensory Profile questionnaire, we checked that all participants had normal hearing ability [61]. The Sensory Profile questionnaire was translated and cross-culturally adapted to Persian, demonstrating proper validity and reliability for assessing Persian-speaking children [59]. According to the Edinburgh Handedness Inventory, all the volunteers were right-handed [62]. The IQ, Conners, Gars, and sensory profile (auditory) scores for both groups are provided in the Supplementary Material.

The Ethics Committee of the IRAN University of Medical Sciences approved the study and all experimental protocols (IR.IUMS.REC.1398.1398). All participants were volunteers, and their parents or guardians declared their consent by filling out the informed consent form.

Also, this study was part of a project at the National Institute for Medical Research Development (NIMAD) in Iran.

### 2.2. Task Design

**Figure 1:**
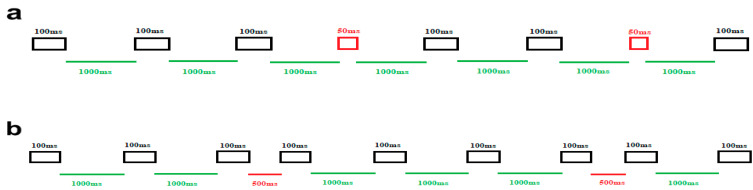
Schematic illustration of the stimuli and experimental setup. (**a**) The upper panel illustrates block 1, focused on duration deviations. The frequent duration stimuli (100 ms) are represented by black rectangles, while the deviant duration stimuli (50 ms) are depicted in red rectangles. ISI is 1000 ms, displayed in green lines. (**b**) The lower panel represents block 2, which is designed around ISI deviations. In this block, the green line signifies the standard ISI (1000 ms), and the red line denotes the deviant ISI (500 ms). All auditory stimuli last for 100 ms, depicted in black rectangles.

To address the primary aim of examining temporal auditory processing in ASD children, a widely used classical timing paradigm featuring two distinct blocked conditions was designed. In one block, our objective was to assess the amplitude and latency of MMN responses induced by duration deviations, while in the other block, the variations focused on the ISI durations. The specific features of the standard and deviant stimuli in these separate blocks were as follows (see Figure 1). In block 1, the duration of auditory stimulus was considered the target of the task, so frequent stimuli were presented for 100 ms in duration (black rectangles), while deviant stimuli were presented for 50 ms (red rectangles). The ISI in this block did not change and remained fixed (1000 ms) (green lines) (Figure 1a). In block 2, the aim of including this block was to elicit MMN responses by manipulating the ISI. Here, the duration of all stimuli was 100 ms (black rectangles). Nevertheless, the standard ISI (green lines) and the deviant ISI (red lines) were 1000 ms and 500 ms, respectively (Figure 1b).

In each block, a total of 500 stimuli were presented. Out of these, 80% were standard trials (400 trials), and the remaining 20% were deviant stimuli (100 trials). All stimuli were held at a fixed frequency of 1000 Hz and an intensity of 60 dB, measured using the Decibel X—Pro Sound Meter (SkyPaw Co, Ltd., Bangkok, Thailand). The presentation order of standard and deviant stimuli in both blocks was pseudo-randomized, ensuring that there was always at least one standard stimulus between every two consecutive deviant stimuli. The order of block presentation was counterbalanced. Each block of the experiment took approximately 10 min to complete, and the entire task lasted around 30 min, accounting for a brief rest period between the two blocks. The total time required for the entire experiment, including subject preparation, was approximately one hour.

### 2.3. EEG Acquisition and Analysis

Electroencephalogram (EEG) was measured using a Mitsar Cap (10–20 international system), with linked ears as reference. Standard 19-channel easy-cap, Ag/AgCl surface electrodes were placed on the participant’s head based on the standard 10–20 system. The EEG was recorded continuously with a sampling rate of 250 Hz, with AFZ as the ground electrode, and filtered online from 0.53 to 50 Hz. The impedance was kept below 10 kilo-ohm. Participants were comfortably seated in a chair, and the projector screen was positioned at their eye level. Two loudspeakers placed 1 m behind the participant in a sound-attenuated room. Auditory stimuli were delivered to the participants during EEG recording while watching a silent animation.

Offline analyses were performed using WinEEG (v. 3.4.9). The data were filtered using a 0.53–30 Hz band-pass filter. We carried out an ICA, and components contaminated by artifacts (eye blinks, eye movements, and muscular activity) were removed. Gross artifacts were eliminated through a visual examination conducted by an experienced individual without any prior knowledge concerning the origin of the data. Subsequently, the data were epoched from 100 ms pre-stimulus to 500 ms post-stimulus. For baseline correction, the epochs were deducted from the mean value of the pre-stimulus interval. Trials with contamination in more than 50% of channels were excluded. Any remaining epochs containing significant physiological artifacts (amplitude exceeding ±75 μV) in any of the 19 channel sites were rejected from further analyses.

Finally, only artifact-free epochs were averaged along with trials and participants for the ERP analysis. The MMN latency was determined by identifying the time point where 50% of the area of the MMN trough was distributed on each side. To conduct this analysis, we focused on the difference wave between the initial and final points where the amplitude reached at least half of the MMN amplitude [63]. It is important to note that this analysis was carried out on the MMN waveforms at specific channels (Fz and Cz), in accordance with previous suggestions [64]. This approach aligns with prior research, which has consistently indicated that larger MMN amplitudes tend to be observed at frontocentral electrodes [65,66,67].

### 2.4. Statistical Analysis

The MMN amplitude and latency data were considered as dependent variables. Continuous variables are presented as means and standard deviations. The data were analyzed by a mixed model analysis of covariance consisting of a between-subject factor (“group”: ASD vs. TD) and a within-subject factor (“deviant-type”: duration vs. ISI deviant) while controlling for age. Statistical calculations were analyzed using SPSS 25.0 (SPSS Inc., IBM Corporation, Armonk, NY, USA), with the statistical threshold set at *p* = 0.05, two-tailed.

## 3. Results

As stated in the Participants Section, we matched the TD and autism groups based on age and IQ. To examine the absence of comorbidity between autism and ADHD, we also compared Conners’ scores using the t-test. As presented in Table 1, it can be observed that there are no significant differences in age, IQ, and Conners’ scores between the two groups. Moreover, the GARS scores were compared between the autism and non-autism groups. As expected, a significant difference in GARS scores was observed between the two groups.

**Figure 2:**
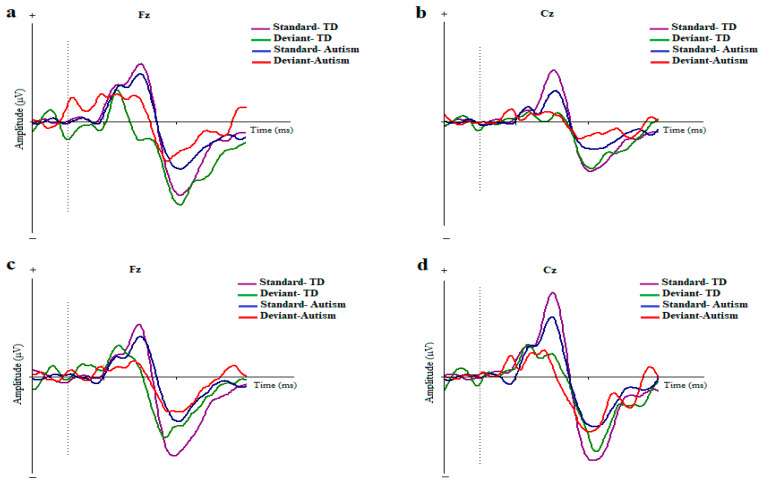
Grand averages of ERP waves to the standard and deviant stimuli in children with autism disorder and TDC at Fz and Cz; Dotted line indicates the start of stimulus (**a**,**b**) Duration-based block. (**c**,**d**) ISI-based block (MMN, mismatch negativity; Fz, frontal electrode; Cz, central electrode).

The original ERPs to the standard and deviant stimuli in the two conditions and for the electrodes are illustrated in Figure 2.

**Figure 3:**
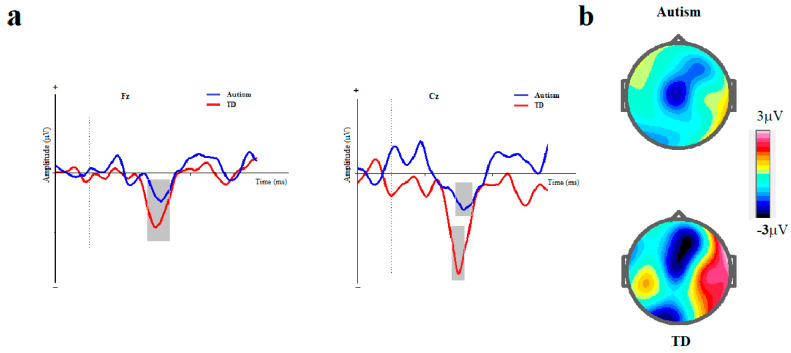
Duration-based block. (**a**) Grand averages of mismatch negativity event-related potentials in children with autism disorder and TDC at Fz and Cz; Dotted line indicates the start of stimulus and grey area represents MMN peak. (**b**) The scalp topographical map for MMN. The top topography corresponds to the autism group, while the bottom one is for the non-autism group (MMN, mismatch negativity; Fz, frontal electrode; Cz, central electrode).

**Figure 4:**
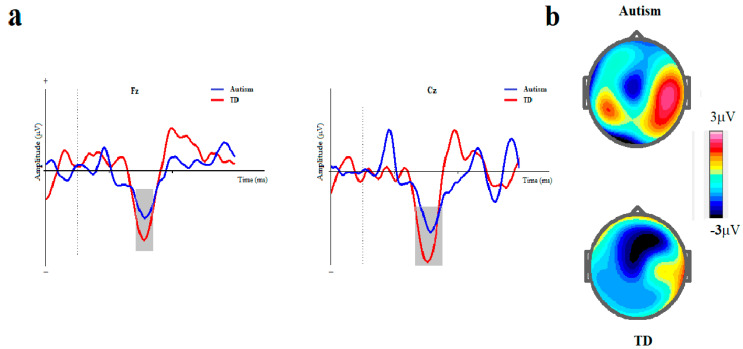
ISI-based block. (**a**) Grand averages of mismatch negativity event-related potentials in children with autism disorder and TDC at Fz and Cz; Dotted line indicates the start of stimulus and grey area represents MMN peak. (**b**) The scalp topographical map for MMN. The top topography corresponds to the autism group, while the bottom one is for the non-autism group (MMN, mismatch negativity; Fz, frontal electrode; Cz, central electrode).

The means and standard errors for amplitude and latency of MMN are presented in Table 2 and ERP waveforms are depicted in Figure 3 and Figure 4.

### 3.1. Comparison of MMN Amplitudes

#### 3.1.1. Fz Electrode

The MMN assessment of the ASD group and TD controls indicated that the ASD group had a significantly lower mean amplitude at Fz than the TD control. The main effect of the group (ASD vs. TD) on MMN amplitude at Fz was marginally significant [F (1, 42) = 4.141, *p* = 0.050, η^2^ = 0.091]. The main effect of the block (duration vs. ISI) on MMN amplitude was not significant ([ F (1, 41) = 1.116, *p* = 0.297, η^2^ = 0.027]). Moreover, the interaction effect of group× block on MMN amplitude was not significant ([F (1, 41) = 0.115, *p* = 0.737, η^2^ = 0.003]).

#### 3.1.2. Cz Electrode

The mixed ANCOVA of MMN showed a lower mean amplitude for ASD than did the TD controls. Notably, the effect of the group on MMN amplitude at Cz was significant [F (1, 41) = 9.871, *p* = 0.003, η^2^ = 0.194]. The main effect of the block (duration vs. ISI) on MMN amplitude was not significant ([F (1, 41) = 0.254, *p* = 0.617, η^2^ = 0.006]). Moreover, interaction effect of group× block on MMN amplitude was not also significant ([F (1, 41) = 0.190, *p* = 0.665, η^2^ = 0.005]).

### 3.2. Comparison of MMN Latencies

#### 3.2.1. Fz Electrode

MMN evaluation indicated that the latency was significantly longer in the ASD group at Fz than in the TD group ([F (1, 41) = 10.381, *p* = 0.002, η^2^ = 0.202]). The main effect of the block on MMN latency was not significant at Fz ([F (1, 41) = 3.656, *p* = 0.063, η^2^ = 0.082]). Moreover, the interaction effect of group× block on MMN latency was not significant ([F (1, 41) = 3.600, *p* = 0.065, η^2^ = 0.081]).

#### 3.2.2. Cz Electrode

MMN evaluation indicated that the latency was significantly longer in the autism group at Cz than in the TD group ([F (1, 41) = 9.979, *p* = 0.003, η^2^ = 0.196]). Furthermore, the main effect of the block on MMN latency was not significant ([F (1, 41) = 0.634, *p* = 0.431, η^2^ = 0.015]). The interaction effect of group× block on MMN latency was not significant ([F (1, 41) = 3.301, *p* = 0.077, η^2^ = 0.075]).

## 4. Discussion

Auditory processes fundamental to social communication are frequently reported to be atypical in children with ASD. The current research investigated the auditory MMN amplitude and latency in response to duration and ISI changes of simple sound stimuli in an oddball task. We employed this MMN paradigm with such duration and ISI deviants in two groups of children with ASD and TD controls. Our results showed that the MMN component reached maximum amplitude in the frontocentral electrodes. We observed a significantly smaller MMN amplitude triggered by both duration and ISI-based deviants in ASD compared to the TD controls. Furthermore, the MMN latency in the ASD children was longer than in the TD children in both blocks. These atypical ERP responses might indicate abnormal processing duration changes in children with ASD with diagnostics potentials.

MMN amplitude changes in response to duration changes of auditory stimuli have been reported in previous oddball paradigms [68,69]. Neurophysiological reports demonstrated that children with ASD exhibited reduced MMN amplitudes in response to differences in tone duration and alterations in vowel duration [30,42]. Additionally, they display diminished MMN responses to subtle temporal indicators like formant transition duration [70]. The decreased MMN amplitude in ASD children might reflect the differential processing of the two different deviant stimuli in our groups. A reduced MMN amplitude in children with ASD suggests that their automatic orienting reflex to deviations is less precise when compared to TD children, a finding that aligns with previous reports [71,72]. Reduced MMN amplitudes could demonstrate a relative hyposensitivity to auditory stimuli in our ASD participants. This is consistent with the suggestion that individuals with ASD may typically exhibit more of a tendency towards sensory stimuli hypo-responsiveness rather than hyper-responsiveness [48,49,73]. From a neural perspective, hyper-responsiveness and hypo-responsiveness are terms used to describe elevated or reduced neural firing, respectively [4].

In this regard, our findings regarding smaller MMN indicate reduced neural firing in response to deviant durations. In line with our findings, it is reported that hypo-responsiveness might be considered the most distinguishing factor between children with ASD and those with developmental delay and TD [74]. This implies that a pattern of diminished response may be the most distinct pattern of ASD, supported by a meta-analytic review [75].

Interestingly, in a study involving high-risk infants, it was observed that those who later developed ASD exhibited a higher prevalence of auditory symptoms and hypo-responsive patterns when compared to high-risk infants who did not develop ASD and low-risk infants [76]. Additionally, hypo-responsiveness has been suggested to be linked to diminished levels of social communication, a trait commonly observed in individuals with ASD [48,77,78]. The findings regarding MMN latency in autism are inconsistent, as some studies indicate a normal MMN response in individuals with ASD. For example, no significant difference in MMN latency in high-functioning ASD individuals was observed manipulating the complexity of tonal and vowel stimuli [79]. Similarly, Kemner et al. (1995) did not find abnormalities in speech-sound-induced MMN in children with autism [51].

However, consistent with the results of the present study, evidence for an abnormal latency MMN response in ASD was demonstrated, reporting delayed MMN following simple tones in ASD [80]. In addition, Oram-Cardy et al. (2005) found that children with ASD and language impairment had delayed MMN responses to both vowel and tone contrasts. Nevertheless, this relatively longer latency was not specific to speech but may be a more general feature of children with ASD [81]. Our findings suggest that the time course of auditory mismatch in terms of stimuli and ISI duration changes can also be considered a neural index of the auditory system in ASD.

Some differences in the results of mismatch autism studies are likely due to the different methods and sample characteristics used in each research. In contrast to findings that reported hyper-responsiveness [82] or intact early sensory sound processing [79], the matched sample in the present study mitigates the intrinsic heterogeneity that is well-known in studies of ASD [83,84]. Other differences in the study design may also explain why our results differ from those of previous studies. For example, others used frequency changes in tone stimuli to elicit MMN responses and found that the MMN latency was shortened [85]. This difference in the direction of the latency effect may be due to the use of different deviants in the oddball paradigm. Furthermore, findings of intact MMN latency following speech-sound differences in nine high-functioning ASD children in the study of Čeponienė et al. might arise from several differences as follows: 1—a smaller sample size compared to our study; 2—not controlling for other factors such as IQ; 3—considering different deviant characteristics; or 4—a combination of these factors. In addition, they have used language-related speech sounds paired with stimulus rendered deviant by manipulating formant frequencies. This method of using deviants is quite different from the duration deviant method employed here. Hence, it is difficult to directly compare our MMN results with other findings [79]

Moreover, the relatively smaller and significantly longer MMN amplitudes in duration and ISI-based stimuli align with reports demonstrating ASD deficits in temporal auditory discrimination processes [86]. Evidence from various cognitive domains indicates that individuals with ASD tend to perform less effectively on tasks that involve temporal processing demands. For example, ASD children exhibit lower performance on intelligence measures incorporating a temporal dimension or requiring sequential processing than assessments involving static stimuli and simultaneous presentation [87,88]. Sensitive temporal processing and attending to temporal information are crucial for recognizing the temporal coordination of shared actions involving objects in the surroundings. This coordination forms the foundation for the emergence of social concepts like the theory of mind [89]. Children later identified as ASD have exhibited difficulties in social referencing. This includes significantly reduced instances of gazing at their caregiver when faced with uncertainty and not effectively using the information provided by the caregiver to influence their actions [90,91]. Considering that socio-cognitive deficits are a hallmark of ASD, it is possible that an early emerging deficiency in temporal processing, as reflected by duration and ISI-based deviants, might play a potential role in disrupting typical social development in this population. One diagnostic criterion for ASD in the DSM-5 involves hyper- or hypo-reactivity to sensory input, or unusual interest in sensory aspects of the environment [3]. This particular symptom was included in the DSM after numerous studies revealed that individuals with ASD, spanning various age groups, frequently display heightened or reduced sensitivities to sensory inputs [48,92,93,94]. It is suggested that sensory sensitivities might play a role in, or perhaps even be a fundamental symptom of, ASD [95]. Such impairments, such as abnormalities of the MMN in our findings, can give rise to communication problems. This obstacle may lead to increased social communication deficits, as evidenced by the positive association between the number of sensory sensitivities and the severity of ASD in children [96]. Also, since such differences in our research were observed for pure sounds, this might account for domain generality in atypical auditory processing in autism. The findings suggest that the perceptual challenges in ASD are not limited to language-specific stimuli but extend to general auditory processing [52]. This domain-general deficit indicates that individuals with ASD might have a broader impairment in how they process and respond to a variety of auditory inputs, which could affect multiple areas of cognitive and social functioning. Such a generalized auditory processing deficit may underlie the sensory sensitivities observed in ASD, contributing to difficulties in communication and social interactions by disrupting the ability to accurately perceive and interpret auditory information from the environment. However, it is important to consider another potential interpretation regarding domain-specific versus domain-general auditory processing deficits. Some deficits might be language-dependent for specific acoustic features [97]. For example, pitch processing deficits in autistic tonal-language speakers have been found to be limited to speech stimuli, suggesting a language-specific issue [98]. Additionally, the complexity of sound stimuli might play a role. In this study, only simple sounds were used, and the results could differ with the use of complex sounds. Hypothetically, if duration differences were examined using speech stimuli, the MMN differences between the ASD and control groups might disappear, indicating that the neural sensitivity to duration differences might be limited to simple nonspeech stimuli rather than representing a domain-general issue. Future studies should incorporate a variety of auditory stimuli, including complex sounds and speech, to further explore such issues.

Moreover, these sensory abnormalities might represent one of the initial observable indicators of ASD [99]. Consequently, in addition to existing diagnostic criteria that predominantly emphasize social abilities, focusing on sensory processing can enable clinicians to diagnose individuals earlier. The emphasis on investigating sensory processing might be obtained using simple tasks, such as duration oddballs, without explicit requirements. Indeed, even though atypical social behaviors typically emerge between 6 and 12 to 18 months of age, ASD is usually diagnosed around 3 to 4 years of age [100]. This is because social behaviors are challenging to detect or describe in children at younger ages [101,102]. In contrast, auditory sensory abnormalities obtained with electrophysiological methods such as ERPs can be assessed even in infancy [103,104,105,106]. Thus, researchers and clinicians can adopt this information at an earlier age, initiating treatment sooner as earlier intervention is linked to more favorable outcomes [107]. However, it is important to establish the robust reliability of MMN measurements at the individual subject level before considering them as a diagnostic tool within the RDoC framework. This entails conducting research to determine the consistency and repeatability of MMN responses across different contexts and time points for individual subjects. Such reliability studies are crucial to ensure that MMN can serve as a dependable biomarker for ASD. Furthermore, normative data and standardized protocols must be developed to account for individual variability and potential confounding factors. Until then, while MMN remains a promising research tool, its application in diagnostic settings should be approached with caution.

The present work has several limitations that warrant consideration. Firstly, the participants included in our study were children with ASD who did not exhibit significant intellectual and motor impairments, tic disorders, or stereotypies. Therefore, our findings may not generalize to children with ASD who present with these additional conditions or symptoms. Secondly, the age range of our participants might introduce variability in developmental aspects. This variability could potentially impact the interpretation of age-related changes in the MMN component across the ASD and TD groups. Additionally, while the present study focused on investigating early duration processing using an oddball paradigm, future research could benefit from employing age-related analyses to better capture developmental changes in processing abilities. Lastly, the findings presented here are based on an ERP methodology that requires passive engagement from participants. Although this approach allows for the investigation of neural responses to auditory stimuli, it may not fully capture the complexities of auditory processing as experienced in naturalistic environments or dynamic social interactions. The present work also did not include other auditory tasks or other ERP measures (such as N1 or P3a) that might be important for establishing baseline auditory processing capabilities. Including these measures in future studies will provide a more comprehensive assessment of auditory processing in ASD and help determine whether the observed differences in MMN are specific to the stimuli used or reflect a more generalized auditory processing difference in the ASD group.

## 5. Conclusions

To our knowledge, this is the first study investigating early duration processing considering duration and ISI changes in stimuli. The current research findings were based on an oddball paradigm that required passive responses from the participants. Our findings suggest that such patterns of neural responses could potentially serve as risk factors for the development of ASD and point to the importance of sensory processes for the identification of the disorder within the RDoC framework [108]. By investigating the neural responses associated with auditory processing in children with ASD, our findings contribute to the RDoC framework objective of elucidating the neural mechanisms underlying specific domains, such as sensory processing abnormalities, that may contribute to the development and manifestation of psychiatric disorders. This approach allows for a more comprehensive examination of ASD beyond traditional diagnostic categories, providing insights into the underlying neural systems involved in sensory atypicality. Furthermore, emphasizing sensory processes within the RDoC framework acknowledges the importance of examining these fundamental domains as potential contributors to the etiology and clinical presentation of ASD. Understanding how alterations in sensory processing, as evidenced by our study findings, intersect with other domains outlined in the RDoC matrix (such as perception, cognition, and social processes) could facilitate obtaining additional insights into ASD neurobiological underpinnings. Finally, we propose that this collective approach might pave the way for the development of novel assessment tools, early intervention strategies, and potentially more targeted treatments, all within the framework guiding principles.

## Figures and Tables

**Table 1:** Descriptive statistics for age, IQ, Conners, and Gars scores, for two groups.

Variable	Group	Mean ± SE	t	Df	* *p* Value
Age	TD	7.125 ± 0.39	0.814	42	0.420
	Autism	6.6 ± 0.52			
IQ	TD	103.96 ± 1.21	0.978	42	0.334
	Autism	102.1 ± 1.488			
Conners	TD	41.63 ± 1.47	−1.135	42	0.263
	Autism	44.10 ± 1.60			
Gars	TD	24.58 ± 2.33	−13.153	42	0.001
	Autism	62.95 ± 1.54			

* Two-sided *p* value < 0.05; values are presented as mean ± SE.

**Table 2:** Descriptive statistics for mismatch negativity potential amplitudes and latencies in both blocks.

	Variable	TD Group (*n* = 25)	ASD Group (*n* = 25)
Duration-based Block	MMN Amplitude (μV)		
	Fz	−1.870 ± 0.329	−0.976 ± 0.360
	Cz	−2.256 ± 0.399	−0.822 ± 0.437
	MMN Latency (ms)		
	Fz	226.25 ± 5.986	256.00 ± 6.558
	Cz	225.17 ± 6.018	254.00 ± 6.593
ISI-based Block	MMN Amplitude (μV)		
	Fz	−2.074 ± 0.322	−1.472 ± 0.353
	Cz	−2.735 ± 0.443	−1.753 ± 0.485
	MMN Latency (ms)		
	Fz	212.46 ± 4.254	221.80 ± 4.660
	Cz	217.08 ± 5.162	221.40 ± 5.654

Values are presented as mean ± SE.

## Data Availability

The data that support the findings of this study are available from the corresponding author upon reasonable request. The data are not publicly available due to privacy restrictions.

## Supplementary Materials

The following supporting information can be downloaded at: https://www.mdpi.com/article/10.3390/brainsci14090896/s1, Table S1: IQ, Conners, Gars, Sensory profile (auditory part) test scores in children with and without Autism.
