# Supplementary material for: Early Auditory Temporal Processing Deficit in Children with Autism Spectrum Disorder: The Research Domain Criteria Framework
Source: Brain Sci. 2024 Sep 3;14(9):896. doi: 10.3390/brainsci14090896 (PMC11430892; doi:10.3390/brainsci14090896)
Supplement: Supplementary file 1 [file brainsci-14-00896-s001.zip › brainsci-3082757-supplementary.pdf]

Table S1. IQ, Conners, Gars, Sensory profile (auditory part) test scores in children with and without Autism.

Group	IQ	Conners	Gars	Sensory Profile (Auditory)
**Normal**	105	49	16	22
**Normal**	106	45	34	20
**Normal**	108	49	19	22
**Normal**	108	46	44	12
**Normal**	110	47	25	10
**Normal**	106	42	14	22
**Normal**	108	48	40	13
**Normal**	92	43	31	13
**Normal**	100	49	4	15
**Normal**	111	30	5	18
**Normal**	107	44	26	11
**Normal**	101	37	26	16
**Normal**	112	36	9	10
**Normal**	113	28	49	21
**Normal**	95	32	15	11
**Normal**	96	41	21	10
**Normal**	101	29	30	18
**Normal**	98	50	25	15
**Normal**	102	46	35	12
**Normal**	110	42	22	21
**Normal**	109	43	27	11
**Normal**	99	50	23	11
**Normal**	102	30	17	17
**Normal**	96	43	33	10
**Autism**	106	48	56	22
**Autism**	106	36	76	20
**Autism**	107	42	55	10
**Autism**	90	50	74	8
**Autism**	106	50	56	22
**Autism**	106	49	59	21
**Autism**	104	48	60	11
**Autism**	106	49	70	20
**Autism**	86	45	67	14
**Autism**	102	28	58	8
**Autism**	107	49	70	23
**Autism**	104	47	56	22
**Autism**	98	46	64	11
**Autism**	97	35	57	17
**Autism**	95	48	68	15
**Autism**	94	29	69	21
**Autism**	111	48	55	11
**Autism**	104	51	70	16
**Autism**	110	48	60	15
**Autism**	103	36	59	21
